# Preparation, Oral SNEDDS Formulation, and In Vivo Evaluation of the HIV-1 Latency-Reversing Agent EK-16A

**DOI:** 10.3390/molecules31111897

**Published:** 2026-06-01

**Authors:** Lu Jin, Yuqi Zhu, Fan Yang, Ting Chen, Xinyi Yang, Yuan Tang, Yipeng Cheng, Dengji Zhang, Jingna Xun, Jun Liu, Bin Wang, Chunyu Li, Xingyu Wang, Suixiang Li, Xingwen Yu, Zhujian Wang, Yiping Zhang, Qian Zhong, Jianrong Ma, Jing Xue, Huanzhang Zhu

**Affiliations:** 1 School of Life Sciences, Fudan University, Shanghai 200438, China; 19110700151@fudan.edu.cn (L.J.); zhuyuqi@fudan.edu.cn (Y.Z.); xinyy@fudan.edu.cn (X.Y.); 23110700149@m.fudan.edu.cn (Y.T.); 24110700006@m.fudan.edu.cn (Y.C.); zhdengji518@163.com (D.Z.); xunjingna@163.com (J.X.); liujun@fubio.cn (J.L.); 20110700164@fudan.edu.cn (B.W.); 23210700131@m.fudan.edu.cn (C.L.); 23110700068@m.fudan.edu.cn (X.W.); 24210700030@m.fudan.edu.cn (S.L.); 23210700174@m.fudan.edu.cn (X.Y.); wangzhujian@163.com (Z.W.); 25110700215@m.fudan.edu.cn (Y.Z.); 25110700153@m.fudan.edu.cn (Q.Z.); 2 College of Clinical Pharmacy, Shanghai Jiao Tong University School of Medicine, Shanghai 200127, China; bill1985@126.com; 3 NHC Key Laboratory of Human Disease Comparative Medicine, National Center of Technology Innovation for Animal Model, Institute of Laboratory Animal Science, Chinese Academy of Medical Sciences and Peking Union Medical College, Beijing 100021, China; chent@cnilas.org (T.C.); majianrong@pumc.edu.cn (J.M.);

**Keywords:** *Euphorbia kansui*, HIV-1, EK-16A, SNEDDS, PI3K/Akt

## Abstract

**Background/Objectives**: AIDS is a serious threat to human health and remains incurable; however, EK-16A, an ingenol derivative, shows promise as a functional cure. In this study, we aimed to extract EK-16A from *Euphorbia kansui*, used in traditional Chinese medicine, to develop an oral self-nanoemulsifying drug delivery system (SNEDDS) for EK-16A and evaluate it in vivo. **Methods**: EK-16A was purified by SFC combined with conventional extraction. The optimal SNEDDS formulation was selected by emulsification and stability testing. Pharmacokinetics, metabolomics, and proteomics were used for in vivo evaluation. **Results**: 1. The extraction yield of EK-16A was four times higher than that of the conventional process; the extraction scale was increased by 25 times, and the purity of EK-16A reached 98.0%. 2. EK-16A is a BCS Class IV compound with low solubility and permeability. The compound’s content degraded to 49.8% after 3 months at 25 °C/60% RH. The EK-16A SNEDDS formulation A#1 showed no degradation after 3 months at 40 °C/75% RH. The absolute bioavailability after oral administration of formulation A#1 in rats was 0.445%. 3. The proteomics results showed that EK-16A significantly downregulated the PI3K-AKT signaling pathway in SHIV-infected rhesus macaques. Specifically, all 11 identified differential proteins were significantly downregulated. **Conclusions**: 1. The extraction process for EK-16A features high yield, purity and large scale. 2. The SNEDDS formulation enhances the stability of EK-16A and successfully delivers this low-solubility and permeability compound into the systemic circulation. 3. Proteomics analysis revealed that EK-16A significantly downregulates the PI3K-AKT signaling pathway in SHIV-infected rhesus macaques. However, further experiments, such as measuring plasma viremia and cell-associated SHIV RNA, are needed to confirm this mechanism.

## 1. Introduction

Acquired immunodeficiency syndrome, known as AIDS, is a systemic disease caused by the human immunodeficiency virus (HIV). The US Centers for Disease Control first reported AIDS in 1981, and over 40 million people have died from the disease since its discovery [1]. HIV can be classified as HIV-1 or HIV-2 [2], with the former accounting for over 95% of infections and causing the worldwide pandemic. The immunodeficiency resulting from HIV infection facilitates the spread of numerous other infectious diseases across countries and entire regions [2,3]. Currently, antiretroviral therapy (ART) is widely used, but it has many side effects [4,5,6,7,8,9] and cannot cure the disease. The reason for this is that HIV-1 latently resides in reservoirs within the body; when ART is stopped, the virus rebounds and replicates [10,11,12,13,14,15,16,17]. Even after decades of continuous ART, latent reservoirs persist, leading to lifelong infection [18,19,20].

Therapeutic approaches for eliminating the HIV latent reservoir include Kill Augmentation, stem cell gene therapy, gene editing, and the “shock and kill” strategy, among others. Kill Augmentation strategies include NK cell therapy, chimeric antigen receptor T-cell therapy (CAR-T), and broadly neutralizing antibody (bNAb) therapy targeting the HIV envelope protein [21,22,23,24]. A typical example of stem cell gene therapy is the transplantation of CCR5-∆32 donor stem cells [25]. Gene editing technologies include CRISPR/Cas9 [26], which can be used to modify host cells.

The “shock and kill” strategy, a widely studied approach, uses latency-reversing agents (LRAs) to activate latent HIV-1, followed by elimination of the virus through the host’s immune system or ART [27,28,29,30,31,32].

Our research group previously screened and identified EK-16A, an ingenol diterpenoid compound and highly active LRA found in *Euphorbia kansui*, used in traditional Chinese medicine. Its ability to reactivate HIV in C11 and J-Lat 10.6 latent cell models is over 200 times higher than that of the commonly used PKC agonist Prostratin and more significant than that of 21 other compounds found in E. kansui [33]. Because EK-16A can act on both HIV-1 transcription initiation (releasing NF-κB) and transcription elongation (promoting P-TEFb expression), it achieves a high activation efficiency [33]. EK-16A has been confirmed to display significant reactivation effects in various HIV-1 latent mouse models [34,35].

The extraction of EK-16A from *Euphorbia kansui* has historically been hampered by issues including low yield, small batch size, and a lack of purity and quality characterization [36,37,38]. In this study, we developed an efficient, environmentally friendly extraction process using supercritical fluid chromatography (SFC)—which achieves a high yield and purity, and has a large batch capacity—to comprehensively characterize the purity of EK-16A.

For AIDS patients, oral medications entail better compliance than injectable ones. However, EK-16A has low solubility and permeability; thus, it is classified as a Biopharmaceutics Classification System (BCS) Class IV compound. It is unstable at room temperature, making the development of oral formulations challenging. SNEDDS is an advanced formulation technology that can improve drug solubility, permeability, and stability [39,40,41,42,43,44,45,46,47,48]. Therefore, in this study, we developed an oral SNEDDS EK-16A formulation.

Through metabolomic and proteomic experiments, we identified a series of biomarkers in rhesus macaques following SHIV infection and after EK-16A administration, providing a reference for human clinical trials. Interestingly, when the proteomics data were analyzed using the KEGG database, we found that EK-16A significantly downregulates the PI3K-AKT signaling pathway. Numerous studies have demonstrated that inhibition of the PI3K-AKT signaling pathway is a key factor in suppressing HIV-1 entry into host cells [49,50,51,52,53,54,55,56,57]. Therefore, we preliminarily propose that EK-16A may exert a dual mechanism: reactivation of the HIV-1 latent reservoir and inhibition of HIV-1 cell entry.

## 2. Results

### 2.1. Results of the Extraction Process and Quality Characterization of EK-16A Raw Material

The EK-16A compound was first discovered in Euphorbia cyparissias in 1981 [36] and was first isolated from *Euphorbia kansui* in 1992 [37], with its structure identified using 1H-NMR, 13C-NMR, 2D COSY, and mass spectrometry [36,37,38,58]. However, the extraction scale was small, yield was low, and the physicochemical properties and quality were not characterized.

This study initially employed a classical extraction method: 200 kg of E. kansui was extracted with ethanol, followed by ethyl acetate extraction, normal-phase silica gel separation, and reversed-phase preparative separation, yielding 32 g of product with 66.4% EK-16A purity. This product was then processed using efficient and environmentally friendly supercritical fluid chromatography (SFC) separation, which took only 6.5 min, achieving 90.1% purity and yielding 14.5 g of EK-16A. Further purification by reversed-phase HPLC yielded 9.2 g of EK-16A with 98.0% purity.

The yield of EK-16A was 4.6 × 10^−5^, which is 35 times higher than values reported in the literature and four times higher than the yield from our laboratory’s 2015 patent (CN106928063A). Also, the production scale was 460 times larger than that reported in the literature and 25 times larger than that in the patent (CN106928063A) [36,37,38,58].

In addition to the structural identification methods used in the literature, we further performed DEPT, NOESY, HSQC-DEPT, HMBC, TOCSY, and high-resolution mass spectrometry (HRMS) analyses to confirm that the EK-16A structure yielded was consistent with that predicted.

**Figure 1:**
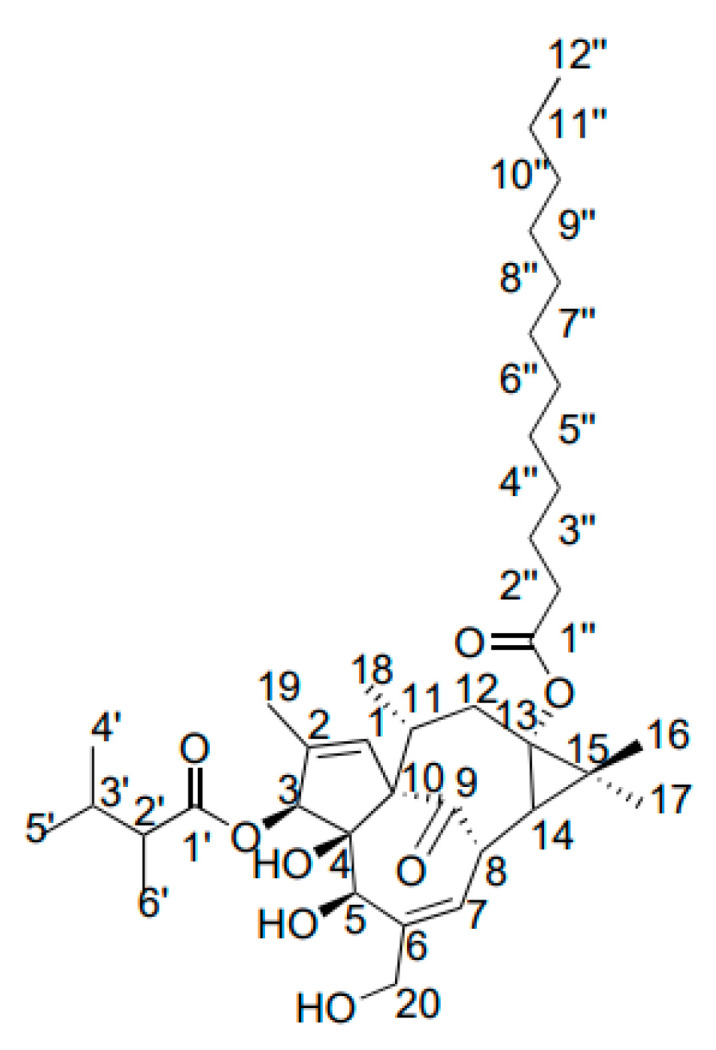
Structural formula of 3-O-(2, 3-Dimethylbutanoyl)-13-O-dodecanoylingenol (EK-16A).

EK-16A’s chemical name is 3-O-(2,3-Dimethylbutanoyl)-13-O-dodecanoylingenol, and its molecular formula is C_38_H_60_O_8_, with a molecular weight of 644.4288. The stereochemical name is (2S,5R,5aS,6S,8aS,9R,10aS)-6-((2,3-dimethylbutanoyl)oxy)-5,5a-dihydroxy-4-(hydroxymethyl)-1,1,7,9-tetramethyl-11-oxo-1,1a,2,5,5a,6,9,10-octahydro-10aH-2,8a-methanocyclopenta[a]cyclopropa[e][10]annulen-10a-yldodecanoate. Its structural formula is shown in Figure 1.

The physicochemical properties and quality characterization results for EK-16A are shown in Table 1. There was a high content of EK-16A compound and it showed few impurities, consistent activity, and structural integrity.

The extraction process for EK-16A developed in this study achieves a high purity, batch yield, and efficiency, while also considering environmental protection. Therefore, it is promising for larger-scale production and application in clinical and GLP toxicology studies.

The EK-16A compound was stable at 2–8 °C for 3 months. When stored at 25 °C/60% RH for 3 months, the content decreased to 49.8%. At 40 °C/75% RH for 3 months, the content decreased to 2.4% (Table 2). EK-16A should be stored at 2–8 °C or lower for long-term preservation. According to the results shown in Table 1 and Table 2, EK-16A is poorly soluble in water and is unstable at room temperature.

### 2.2. Results of the EK-16A Oral Self-Nanoemulsifying Drug Delivery System (SNEDDS) Study

#### 2.2.1. EK-16A Exhibits Low Permeability in Caco-2 Cells

The Lucifer Yellow rejection assay results were all below 0.1%, indicating the good integrity of the Caco-2 cell monolayer.

The measured Papp for nadolol (low permeability control) was 0.0270 × 10^−6^ cm/s, and for metoprolol (high permeability control), it was 20.7 × 10^−6^ cm/s. The efflux ratio (ER) for digoxin (P-gp substrate) was 134. These values were consistent with our theoretical expectations.

The mean apparent permeability coefficient (Papp) of EK-16A in the apical-to-basolateral (A-B) direction was <0.270 × 10^−6^ cm/s, which is below the low permeability threshold of 0.600 × 10^−6^ cm/s. The Papp in the basolateral-to-apical (B-A) direction was <0.243 × 10^−6^ cm/s, also below the low permeability threshold. Thus, EK-16A exhibits low permeability in Caco-2 cells.

#### 2.2.2. EK-16A Exhibits High Solubility in Oily Excipients

Our research group previously demonstrated that EK-16A has good solubility in oil phase vehicles such as ethyl oleate, isopropyl myristate, caprylic/capric mono-diglycerides, caprylocaproyl polyoxylglycerides, and oleoyl polyoxyl-6 glycerides, and in emulsifiers like Kolliphor RH40, Capmul MCM EP/NF, Labrasol, Tween-20, and PEG-400 [35].

Building on these findings, we screened more excipients to promote solubility and absorption, measuring solubility with HPLC. The results showed that EK-16A is highly soluble in several oily excipients, such as Labrasol ALF, Labrafac MC60, Capryol 90, and Maisine CC (Table 3).

#### 2.2.3. Formulation Screening for EK-16A SNEDDS

EK-16A has poor water solubility and permeability; thus, it is classified as a BCS Class IV drug, which presents significant challenges for oral formulation development. SNEDDS is an advanced technology that can enhance drug solubility and permeability and improve formulation stability [39,40,41,42,43,44,45,46,47,48]. Based on the solubility of EK-16A in various oils and emulsifiers, combined with SNEDDS principles and the properties of various excipients, 15 formulations were designed. For example, medium-chain fatty acid esters like Capryol 90 [59] and Labrafac MC60 [60] were used to increase intestinal permeability, while long-chain fatty acid excipients like Maisine CC (glyceryl monooleate) were used to promote lymphatic transport [61], which can effectively improve the bioavailability and stability of drugs [62]. The drug loading of 0.2% (*w*/*w*) was chosen to achieve a daily dose of approximately 4.8 mg EK-16A for a 60 kg adult (based on human equivalent dose from mouse efficacy data) with a total fill weight of 2400 mg (4 capsules of 600 mg). The compositions of these 15 formulations are shown in Table 4.

First, the appearance of the 15 blank formulations (without EK-16A) and their emulsification performance in purified water were observed. Formulation #A-9 was semi-solid, #A-10 was paste-like, and #A-7 failed to disperse and emulsify in water (see Table 5). The remaining 12 formulations were then prepared as drug-loaded formulations containing EK-16A. Their appearance and emulsification performance in simulated gastric fluid were evaluated. Formulations #A-2, #A-3, #A-5, #A-6, and #A-13 did not disperse or emulsify in gastric fluid. The particle size and PDI of the remaining seven formulations were tested in gastric fluid; also, the emulsification performance, particle size, and PDI in simulated intestinal fluid were investigated. The results showed that #A-4 had a particle size exceeding 1000 nm in gastric fluid and was therefore excluded (see Table 6).

The emulsification performance was graded according to the following scale: A, rapid dispersion/emulsification (<1 min), forming a clear or slightly bluish micro emulsion; B, rapid dispersion/emulsification (<1 min), forming a relatively clear micro emulsion; C, slower dispersion/emulsification (<2 min); D, slow dispersion/emulsification; E, no dispersion/emulsification.

Formulation #A-1 exhibited the best emulsification performance, particle size and PDI in both gastric and intestinal fluids, while the other five formulations showed acceptable performance (see Table 6). Formulations #A-1, #A-8, #A-11, and #A-12 were selected for stability studies under long-term and accelerated storage conditions.

#### 2.2.4. Formulations #A-1 and #A-8 Increased the Stability of EK-16A

**Figure 2:**
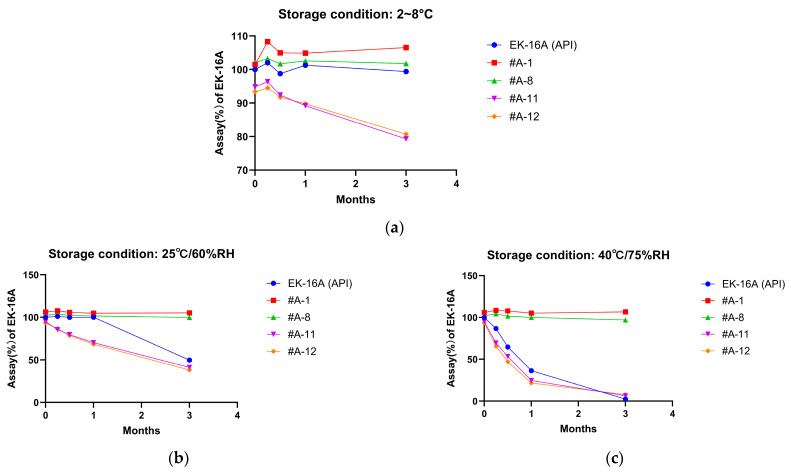
Stability trend of EK-16A compound and oral formulations at 2–8 °C, 25 °C/60% RH, and 40 °C/75% RH. (**a**) Stability at 2–8 °C. (**b**) Stability at 25 °C/60% RH. (**c**) Stability at 40 °C/75% RH.

Formulations #A-1 and #A-8 significantly improved the stability of EK-16A: they remained clear with no content degradation after 1 week, 2 weeks, 1 month, and 3 months under both 25 °C/60% RH and 40 °C/75% RH conditions. In contrast, the other SNEDDS formulations showed varying degrees of degradation. The stability results are shown in Figure 2 and Table 7.

According to Section 2.2.3, formulation #A-1 exhibited better emulsification performance, particle size, and PDI than formulation #A-8 in both gastric and intestinal fluids. Therefore, formulation #A-1 was selected for subsequent in vivo experiments in rats and rhesus macaques.

### 2.3. Results of the In Vivo Pharmacokinetics and Metabolism of EK-16A SNEDDS

#### 2.3.1. The Oral SNEDDS Formulation #A-1 Successfully Delivered EK-16A into Systemic Circulation

(1) The bioanalytical LC-MS/MS method demonstrated good system suitability.

Linear calibration curve: The calibration curve for EK-16A consisted of eight non-zero standards in the concentration range of 0.500–500 ng/mL. The ratio of the peak area of EK-16A to that of the internal standard (IS) celecoxib was defined as Y, and the concentration of EK-16A in plasma was defined as X. Linear regression with a weighting factor of 1/x^2^ was used, and the correlation coefficient (r) was >0.99. The deviation (Bias %) of each calibration standard from the theoretical value was within ±20.0%.

Precision and accuracy: For the determination of EK-16A in SD rat plasma, the LLOQ (1.50 ng/mL) had a precision (CV) of 14.4% and accuracy of 91.3% (n = 6). The precision and accuracy were 8.2% and 94.0%; 9.6% and 93.0%; and 8.2% and 88.2%, respectively, for the low (20.0 ng/mL), medium (200 ng/mL), and high (400 ng/mL) concentrations (all n = 6). These results met the method acceptance criteria (precision CV ≤ 20.0%; accuracy between 80.0 and 120% or Bias within ±20.0%).

Quality control: When analyzing the plasma samples, each sequence included QC samples at four concentrations (1.5, 20, 200, 400 ng/mL) to ensure the accuracy and reliability of the LC-MS/MS system. All QC samples in this study met the acceptance criteria (Bias % within ±20% of the nominal value).

(2) Plasma Concentration–Time Curve and Bioavailability Results for EK-16A Formulations.

**Figure 3:**
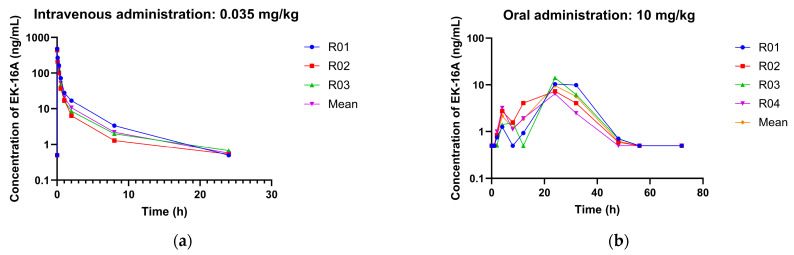
(**a**) Individual and mean plasma concentration–time curves of EK-16A after IV bolus administration of 0.035 mg/kg to SD rats. R01–R03 indicate individual rat numbers, and Mean indicates the average. (**b**) Individual and mean plasma concentration–time curves of EK-16A after oral gavage administration of 10 mg/kg EK-16A SNEDDS formulation #A-1 to SD rats. R01-R04 indicate individual rat numbers, and Mean indicates the average.

Intravenous (IV) bolus injection of EK-16A and oral gavage of EK-16A SNEDDS formulation #A-1 were administered to the test animals. The plasma concentrations and individual and mean plasma pharmacokinetic parameters are shown in Table 8 and Figure 3.

After a single IV bolus dose of 0.035 mg/kg EK-16A in SD rats, the plasma clearance (Cl) was 3.67 ± 0.952 mL/min/kg, the steady-state volume of distribution (Vdss) was 0.787 ± 0.238 L/kg, and the elimination half-life (T1/2) and AUC0-last were 4.97 ± 0.710 h and 163 ± 46.5 ng·h/mL, respectively.

After oral gavage of EK-16A compound at 10 mg/kg in SD rats, EK-16A was not detected in plasma, likely due to in vivo degradation or precipitation.

After oral gavage of the EK-16A formulation (SNEDDS formulation #A-1) at 10 mg/kg in SD rats, the peak concentration (Cmax) of EK-16A was 9.58 ± 3.53 ng/mL, occurring at a Tmax of 24.0 h post-dose; the AUC0-last was 193 ± 45.3 ng·h/mL; and the absolute bioavailability of the 10 mg/kg oral dose was 0.445%.

The bioavailability results indicate that oral SNEDDS formulation #A-1 successfully delivered EK-16A into the rats’ systemic circulation.

#### 2.3.2. Identification of Six Metabolites of EK-16A in Rat Plasma, Feces, and Urine

EK-16A exhibits high in vivo activity, and its metabolites may also contribute to its pharmacological effects. Metabolite identification could also provide new insights for “prodrug design,” such as modifying the parent drug into a more soluble or permeable prodrug form (e.g., phosphorylation, sulfonation) to overcome the solubility and permeability limitations of EK-16A. Therefore, we identified the in vivo metabolites and metabolic pathways of EK-16A.

Metabolite studies were performed using a Bruker tims TOF Pro2 time-of-flight mass spectrometer and the MetaboScape 2024b software (Bruker Daltonics, Bremen, Germany) integrated with BioTransformer. BioTransformer combines knowledge-based and machine learning methods to predict molecular metabolism in human tissues and organs, outperforming other advanced metabolism prediction tools like Meteor Nexus and ADMET Predictor [63]. Following administration of EK-16A injection and oral formulation #A-1 to rats, plasma and excreta samples were collected and analyzed by LC-TOFMS. The MS1 and MS2 spectra of metabolites were monitored and matched with metabolites and pathways predicted by BioTransformer. Six metabolites were identified, all of which are ingenol diterpenoids (Table 9).

The literature indicates that ingenol derivatives with carboxylic ester substituents at the C3 or C13 position have superior HIV-1 latency reactivation effects compared to other ingenol compounds. Ingenol without these substituents has the weakest activity [64]. EK-16A has carboxylic ester substituents at both the C3 and C13 positions, further supporting the finding that its activity is higher than other ingenol compounds from E. kansui.

The experimental results show that EK-16A is metabolized in vivo via hydrolysis by carboxylesterase EC 3.1.1.1 and sulfonation by sulfotransferase EC 2.8.2.2, generating six metabolites. Metabolites 1 and 2 retain a carboxylic ester substituent at the C13 and C3 positions of ingenol, respectively. Metabolite 3 is ingenol, without substituents. All three metabolites have been reported to display HIV-1 reactivation activity, but at a lower level than that of EK-16A, with ingenol showing the weakest activity [64].

Metabolites 4–6 each have a sulfonic acid group added at the C20 position. Their activities have not been reported, but the sulfonic acid group may improve the water solubility of the compound.

The organic synthesis route for EK-16A is complex [64,65]. Interestingly, in this metabolite study, we unexpectedly found that ingenol can be specifically sulfonated at the C20 hydroxyl group by sulfotransferase in vivo, providing a new strategy for modifying the molecular structure of EK-16A to further improve its drug-like properties.

### 2.4. Study on the PI3K/AKT Signaling Pathway of EK-16A in SHIV-Infected Rhesus Macaques

#### 2.4.1. Discovery of Biomarkers in SHIV-Infected Rhesus Macaques Through Metabolomics

Through the metabolomics experiment, we identified 5456 metabolites. Comparing the EK-16A treatment group with the SHIV-infected model group, 293 metabolites were significantly upregulated, and 586 were significantly downregulated. These differential metabolites serve as potential biomarkers for the action of EK-16A in SHIV-infected macaques, and as rhesus macaques are closely related to humans, these results provide cutting-edge data for exploring personalized clinical treatments. Comparing the SHIV-infected latent model group with the healthy control group, 294 metabolites were significantly upregulated and 430 were significantly downregulated, serving as potential biomarkers for latent SHIV infection in rhesus macaques.

#### 2.4.2. Proteomics Study of EK-16A in SHIV-Infected Rhesus Macaques

Proteomic analysis was performed using a Tims TOF HT mass spectrometer (Bruker), DIA (data-independent acquisition) technology, a magnetic nanobead-based method for enriching low-abundance proteins, and Spectronaut Pulsar 18.7 (Biognosys) software for data processing. Bioinformatics methods and tools were used for statistical analysis.

Following LC-MS/MS analysis and qualitative/quantitative analysis, a total of 5232 proteins were identified. This included 4753 proteins in the healthy control group, 4973 in the SHIV-infected model group, and 5050 in the EK-16A SNEDDS treatment group. The molecular weights of the identified proteins were predominantly in the 10–100 kDa range.

##### Differential Protein Expression Analysis

Proteomic data were log10-transformed. Protein expression differences between the groups were assessed using two parameters: Fold Change (FC) and significance (*p*-value). Differentially expressed proteins (DEPs), the overall distributions of which are shown in Table 10, were defined by a *p*-value < 0.05 and FC ≥ 2.0 or FC ≤ 1/2.0.

Comparing the EK-16A treatment group with the SHIV-infected rhesus macaque model group, 123 proteins were significantly upregulated and 110 proteins were significantly downregulated. These differential proteins represent potential biomarkers of EK-16A’s effect on SHIV-infected rhesus macaques; thus, as rhesus macaques are closely related to humans, these findings provide cutting-edge data for clinical trials. Comparing the SHIV-infected rhesus macaque model group with the healthy rhesus macaque control group, 332 proteins were significantly upregulated and 86 proteins were significantly downregulated, representing potential biomarkers for latent SHIV infection in rhesus macaques. There were 63 common differentially expressed proteins between the two comparison groups.

**Figure 4:**
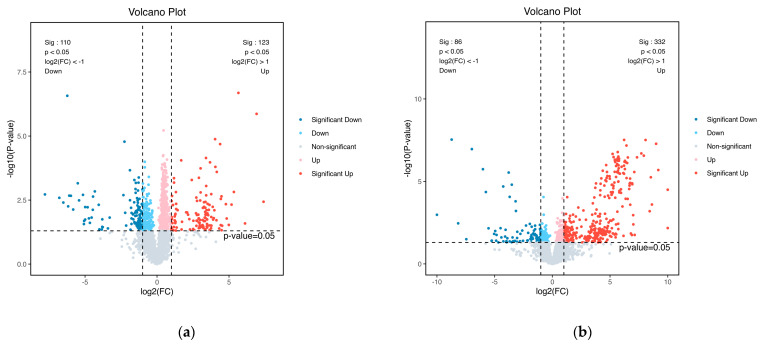
Volcano plots for DEP screening. (**a**) EK-16A vs. Model. (**b**) Model vs. Control. The x-axis is log2 (FC), with points farther from zero indicating greater difference (right: upregulation, left: downregulation). The y-axis is −log10 (*p*-value), with points farther from zero indicating greater significance. Red and blue points represent upregulated and downregulated proteins, respectively, with deeper colors indicating greater significance. Gray points represent proteins with *p*-value ≥ 0.05. Dashed lines represent the thresholds for differential expression: log_2_ (FC) = ±1 (2-fold change) and *p* = 0.05.

Volcano plots provide a visual representation of DEP screening, with red indicating upregulation, blue indicating downregulation, and gray or shaded points representing proteins filtered out (Figure 4).

##### Protein Function Analysis

Bioinformatics databases were used for protein function analysis. Functional enrichment: The background proteins are all proteins in the species with a specific function, while the foreground proteins are the DEPs with that function. A hypergeometric distribution test was used to calculate the *p*-value for significant enrichment.

KEGG Enrichment Analysis: The KEGG database (https://www.genome.jp/kegg/, accessed on 5 April 2026) is an online resource for genomes, enzymatic pathways, and biological chemicals, established by Kanehisa Laboratories in 1995.

Comparing the EK-16A treatment group with the SHIV-infected model group, 233 significant DEPs were enriched into multiple signaling pathways using the KEGG database.

The top 10 pathways with the highest enrichment significance were cytoskeleton in muscle cells; vitamin digestion and absorption; African trypanosomiasis; primary immunodeficiency; glycolysis/gluconeogenesis; PI3K-Akt signaling pathway; B cell receptor signaling pathway; signaling pathways regulating pluripotency of stem cells; HIF-1 signaling pathway; and Fc epsilon RI signaling pathway.

Human immunodeficiency virus type 1 (HIV-1) drives multiple signaling pathways to facilitate its entry and replication. The interaction of the HIV-1 envelope protein (env) with CD4 on the target cell surface first activates the phosphatidylinositol 3-kinase (PI3K)/Akt signaling pathway, which promotes the activation of CCR5/CXCR4 co-receptors. Subsequently, the interaction of the HIV-1 env glycoprotein with CCR5/CXCR4 co-receptors facilitates viral fusion and entry. Therefore, inhibiting the PI3K/Akt signaling pathway is critical for inhibiting HIV-1 entry [49,50,51,52,53,54,55,56,57].

The KEGG enrichment analysis revealed significant downregulation of both the PI3K-Akt and B cell receptor signaling pathways. Eleven foreground proteins (DEPs) in the PI3K-Akt signaling pathway were all significantly downregulated.

**Figure 5:**
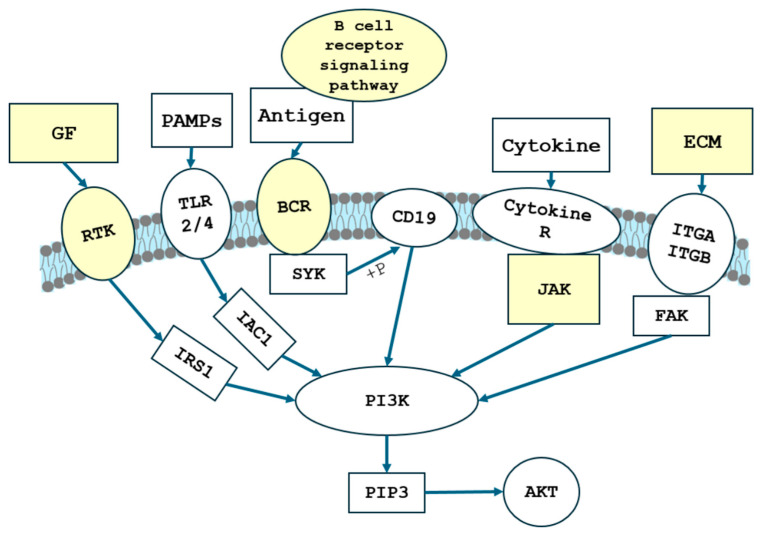
PI3K-Akt signaling pathway diagram (KEGG ID mcc 04151). Following EK-16A administration, the PI3K-Akt signaling pathway was significantly downregulated. Boxes with yellow backgrounds indicate proteins that were significantly downregulated, including GF, RTK, ECM, BCR, JAK, and the B cell signaling pathway.

The PI3K-Akt signaling pathway is initiated by PI3K activation, which then mediates a series of biochemical reactions. There are five pathways that lead to PI3K activation (KEGG signaling pathway diagram, ID mcc 04151), four of which were significantly downregulated, as shown in Figure 5 (highlighted with a yellow background).

GF and RTK downregulation: Significantly downregulated proteins that were enriched include Insulin-like growth factor I (IGF1), Fibroblast growth factor receptor (A0A1D5Q279), and Fibroblast growth factor receptor (F7A4T4). ECM downregulation: Significantly downregulated proteins that were enriched include Reelin Fragment (EGK_14012), Cartilage oligomeric matrix protein (COMP), and Laminin subunit alpha-1 Fragment (EGK_09538). BCR downregulation: Significantly downregulated proteins that were enriched include Ig-like domain-containing protein (A0A5F7ZFP0), Ig-like domain-containing protein (A0A5F8AL51), IGHV3-58*01 Fragment (IGHV3-58), and Anti-quaternary epitope monoclonal antibody heavy chain Fragment (F1D887). JAK downregulation: Tyrosine-protein kinase (LOC114669853) was the only enriched significantly downregulated protein.

Numerous reports indicate that HIV-1 activates the PI3K/Akt signaling pathway to enter host cells, and inhibiting this pathway is crucial for limiting HIV-1 entry [49,50,51,52,53,54,55,56,57]. The proteomics results from this study show that following EK-16A administration, the PI3K-Akt signaling pathway was significantly downregulated, encompassing 11 significantly downregulated proteins. The preliminary conclusion is that EK-16A inhibits HIV-1 entry into host cells by downregulating the PI3K/Akt signaling pathway in rhesus macaques. However, further experiments are needed to confirm this conclusion, such as measuring plasma viremia, cell-associated SHIV RNA, etc.

#### 2.4.3. Molecular Docking Experiments

To investigate the potential targets of EK-16A, molecular docking experiments were performed for the 11 significantly downregulated proteins. Using AlphaFold (version 2), AutoDock Vina (version 1.2.7), and PLIP software (version 2.4.0), the interaction sites (e.g., amino acid residues and specific functional groups of the drug) and interaction energies (including van der Waals forces and electrostatic interactions) between the drug molecule and target proteins were analyzed. The scoring function, Affinity (kcal/mol), represents the binding affinity between the drug and the target. Smaller values indicate stronger affinity, with values < −8 kcal/mol indicating very good affinity; −8 < Affinity < −6 indicates moderate affinity; and Affinity > −6 indicates weak or no affinity.

The molecular interaction between EK-16A and Tyrosine-protein kinase (gene LOC114669853) had an Affinity of −9.688 kcal/mol, indicating a very good affinity. The interaction between EK-16A and Laminin subunit alpha-1 Fragment (gene EGK_09538) had an Affinity of −7.271 kcal/mol, indicating moderate affinity. The interaction between EK-16A and Insulin-like growth factor I (gene IGF1) had an Affinity of −5.061 kcal/mol, indicating weak affinity. The remaining eight proteins showed weak or no affinity for EK-16A, suggesting that their significant downregulation is likely indirect, rather than through direct binding to EK-16A.

The interaction sites for these three proteins with EK-16A are listed in Table 11.

## 3. Discussion

In this study, we report three major achievements. First, we developed an extraction process for EK-16A that achieves high purity, high yield, and large batch size. Second, we formulated the poorly soluble, poorly permeable, and unstable EK-16A into an oral self-nanoemulsifying drug delivery system (SNEDDS), which successfully delivered the compound into systemic circulation and markedly improved its stability. Third, using SHIV-infected rhesus macaques, we discovered that oral administration of EK-16A-SNEDDS significantly downregulates the PI3K-AKT signaling pathway in vivo.

Below, we discuss each finding in detail, along with their implications and directions for future research.

Supercritical fluid chromatography (SFC) is a highly efficient and environmentally friendly chromatographic separation technique [66,67,68]. This study is the first to apply SFC for the extraction of compounds from *Euphorbia kansui*, significantly improving the yield and scale of EK-16A extraction. For the first time, the physicochemical properties and quality of EK-16A have been characterized, including content determination by quantitative NMR (QNMR), residual solvent analysis by GC, chiral purity determination by SFC, and impurity profiling by HPLC. Studies on optical rotation, crystal form, melting point, solubility, and permeability were also conducted, demonstrating that the EK-16A produced by this process is of a high content and has high purity with few impurities. This process is promising for scale-up for future toxicological and clinical studies.

EK-16A is poorly water-soluble, has low permeability, and has poor stability. After 3 months at 25 °C, it degrades to 49.8% of its original content, and after one week at 40 °C, it degrades to 86.8%. Oral administration is challenging due to poor absorption into the systemic circulation. Self-nanoemulsifying drug delivery systems (SNEDDS) present drugs in nano form, enhancing their solubility, permeability, and stability [39,40,41,42,43,44,45,46,47,48]. SNEDDS formulation A#1 developed in this study retained its clear appearance with no content degradation after 3 months at 40 °C/75% RH, significantly improving the stability of EK-16A. The concentration of EK-16A in rat plasma was determined using LC-MS/MS, allowing for the construction of plasma concentration–time curves and calculation of pharmacokinetic parameters. This confirmed that oral SNEDDS formulation #A-1 successfully delivered EK-16A into the systemic circulation.

The oral bioavailability of the SNEDDS formulation is relatively low (0.445%), but due to the high potency of EK-16A, this is sufficient to achieve a pharmacological effect. In our previous study published in *Scientific Reports* in 2017, we demonstrated that the half-maximal effective concentration (EC_50_) of EK-16A in two HIV-1 latently infected cell models (C11 and J-Lat 10.6) was 3.53 nM and 4.06 nM, respectively [33]. After oral administration of a 10 mg/kg dose of the SNEDDS formulation, the peak plasma concentration (Cmax) reached 9.58 ng/mL, which corresponds to approximately 14.9 nM. This concentration is higher than that required for reactivating latently infected HIV-1 cells, indicating that a dose of 10 mg/kg EK-16A is sufficient to achieve the desired pharmacological effect. In future studies, a Design of Experiments (DOE) approach could be employed to further screen for formulations with even higher bioavailability.

To determine whether EK-16A metabolites exhibit activity, they were investigated using a Bruker tims TOF Pro2 time-of-flight mass spectrometer and MetaboScape 2024b software with BioTransformer. The analysis of the rat plasma and excreta samples led to the identification of six metabolites, all belonging to the ingenol diterpenoid class. The activity of metabolites 1–3 has been reported previously [64]. However, that of metabolites 4–6, which feature a sulfonic acid group at the C20 position, has not been reported. From the perspective of prodrug design, modifying the parent compound through phosphorylation or sulfonation can improve solubility [69,70,71]. While the specific substitution of phosphate or sulfonate groups at the C-3, C-5, and C-20 hydroxyl positions of EK-16A is challenging synthetically [64,65], this in vivo metabolism study revealed that rat sulfotransferase can specifically introduce a sulfonic acid group at the C-20 position. This finding provides a promising strategy for drug design and prodrug discovery to improve solubility and permeability.

This study is the first to report the metabolomic profile following EK-16A administration. Our results showed significant upregulation of 293 metabolites and downregulation of 586 metabolites. As rhesus macaques are closely related to humans, these differential metabolites serve as potential biomarkers for EK-16A action, providing critical data for exploring personalized clinical treatments.

Through proteomic analysis of a SHIV-infected rhesus macaque model, this study is the first to demonstrate that EK-16A significantly downregulates the PI3K-Akt signaling pathway in vivo. It is well established that inhibiting the PI3K/Akt signaling pathway is crucial for preventing HIV-1 entry into host cells [49,50,51,52,53,54,55,56,57]. We speculate that EK-16A may have a dual mechanism of latency reversal [33,34,35] and inhibition of HIV-1 entry into cells, but further experiments, such as measuring plasma viremia and cell-associated SHIV RNA, are needed to confirm this hypothesis.

## 4. Materials and Methods

### 4.1. Extraction, Purification, and Quality Characterization of EK-16A

#### 4.1.1. Extraction and Purification of EK-16A

The dried roots of *Euphorbia kansui* (200 kg, purchased from Yuncheng, Shanxi Province, China) were heated under reflux with anhydrous ethanol (300 L) three times, each for 2 h. The resulting combined extracts were concentrated under reduced pressure; the concentrate created was then suspended in purified water (5 L) and extracted three times with an equal volume of ethyl acetate. The combined organic layers were concentrated to 2 L.

The concentrate was mixed with silica gel (2.5 kg), dried under reduced pressure, and loaded onto a normal-phase chromatography column packed with an equal volume of silica gel. The column was eluted stepwise with petroleum ether–ethyl acetate (10:1→5:1→3:1→2:1→1:1). The eluent was monitored using LC-MS, and the fractions eluted with petroleum ether–ethyl acetate = 5:1 to 2:1 were collected, combined, and dried under reduced pressure to yield approximately 438 g of a product designated as GS-EA4. Ether and ethyl acetate were purchased from Tianjin Jindong Tianzheng Fine Chemical Reagent Factory (Tianjin, China).

GS-EA4 (300 mL ethyl acetate solution) was mixed with 450 g of silica gel, dried, and subjected to normal-phase flash column chromatography using an Agela Silica (CS) column (irregular 40–60 μm, 60 Å, 330 g; Agela Technologies, Newark, DE, USA). The mobile phase consisted of petroleum ether–ethyl acetate in a gradient elution at a flow rate of 100 mL/min. The target compound was collected, concentrated, and dried to give 300 g of GS-EA4-5.

This material was dissolved in methanol (600 mL) and further purified using reversed-phase preparative HPLC on a Phenomenex Luna C18 column (300 × 50 mm, 10 μm; Phenomenex, Torrance, CA, USA). The mobile phases were purified water (A) and acetonitrile (B) at a flow rate of 80 mL/min, using the following gradient: 90% B (0–3 min), 90→100% B (5–25 min), 100% B (25–35 min), 100→90% B (35–35.1 min), and 90% B (35.1–40 min). The target fraction was collected, concentrated, and dried to yield 32 g of GS-EA4-5P.

GS-EA4-5P was then separated using supercritical fluid chromatography (SFC) on a Waters SFC 350 Preparative System (Waters Corporation, Milford, MA, USA) using a DAICEL CHIRALCEL OZ column (250 × 50 mm, 10 μm; Daicel Corporation, Tokyo, Japan). The mobile phase was CO_2_/acetonitrile (75/25, *v*/*v*) at a flow rate of 180 g/min over a run time of 6.5 min. The target compound was collected, concentrated, and dried to give 14.5 g of GS-EA4-5PSFC1.

Finally, GS-EA4-5PSFC1 was dissolved in methanol (1.1 L) and subjected to a second reversed-phase preparative HPLC on a Shimadzu LC-20AP system (Shimadzu Corporation, Kyoto, Japan) with a Phenomenex Luna C18 column (300 × 50 mm, 10 μm; Phenomenex, Torrance, CA, USA). The mobile phases were purified water (A) and acetonitrile (B) at a flow rate of 80 mL/min, using the following gradient: 87% B (0–5 min), 87→95% B (5–30 min), 95→98% B (30–33 min), 98% B (33–45 min), 98→100% B (45–45.01 min), and 100% B (45.01–48 min). The target fraction was collected, concentrated, and dried to afford 9.2 g of EK-16A with a purity of 98.0%, as determined by HPLC.

#### 4.1.2. Quality Characterization Method for EK-16A

(1) Structural Identification of EK-16A

Molecular weight was determined using UPLC with time-of-flight high-resolution mass spectrometry (Waters ACQUITY UPLC HCLASS PLUS G2-XS QTOF; Waters Corporation, Milford, MA, USA) with electrospray ionization (ESI). The structure was elucidated using NMR (Bruker 400 MHz; Bruker Corporation, Karlsruhe, Germany), acquiring 1H-NMR, 13C-NMR, DEPT, 1H-1H COSY, NOESY, HSQC-DEPT, HMBC, and TOCSY spectra. The structure was confirmed to be consistent with the theoretical structure.

(2) Optical Rotation (OR)

Optical rotation was measured using a Rudolph AUTOPOL IV polarimeter (Rudolph Research Analytical, Hackettstown, NJ, USA). Light source: Na; detection wavelength: 589 nm; temperature: 20 °C. EK-16A was prepared at a concentration of 1.0014 mg/mL. Specific rotation was calculated using the following formula: [α] = 100α/lc.

(3) Differential Scanning Calorimetry (DSC)

DSC was performed using a Mettler Toledo DSC3 (Mettler Toledo, Columbus, OH, USA). Temperature range: 30.0~400.0 °C; heating rate: 10.00 K/min; nitrogen flow rate: 60.0 mL/min.

(4) Thermogravimetric Analysis (TGA)

TGA was performed using a Mettler Toledo TGA2 (Mettler Toledo, Columbus, OH, USA). Heating rate: 20.0 K/min; temperature range: 40.0~800.0 °C; N2 flow rate: 70.0 mL/min.

(5) Ultraviolet (UV) Absorption Spectroscopy

EK-16A was dissolved in acetonitrile to a concentration of 0.735 mg/mL. UV spectrum was measured using a SHIMADZU UV-2600i instrument (Shimadzu Corporation, Kyoto, Japan) with a scanning wavelength range of 190–800 nm.

(6) Infrared (IR) Absorption Spectroscopy

The IR spectrum was obtained using a Thermo NICOLET iS10 spectrometer (Thermo Fisher Scientific, Waltham, MA, USA) with a scanning range of 600–4000 cm^−1^. The EK-16A powder was placed directly onto the ATR crystal surface.

(7) Powder X-ray Diffraction (XRPD)

XRPD was performed using a Haoyuan DX-2700BH diffractometer (Haoyuan Instrument, Dandong, China). Conditions: 40 kV, 40 mA; slit: 1.0/0.3/1.0; step size: 0.02°; target: Cu; scan range: 3.00–40.00°. An appropriate amount of sample was loaded onto a single-crystal silicon plate for scanning.

(8) Elemental Analysis (EA)

An appropriate amount of EK-16A raw material was combusted, decomposed, and quantitatively converted. The percentages of C, H, and N were determined using an Elementar Automatic Element Analyzer (Elementar Analysensysteme GmbH, Langenselbold, Germany).

(9) Water Content

Water content was determined by coulometric Karl Fischer titration using a Metrohm 852 titrator (Metrohm AG, Herisau, Switzerland) with an oven sample processor.

(10) Content of Assay

The content of EK-16A was accurately determined using Bruker QNMR (quantitative NMR; Bruker Corporation, Billerica, MA, USA).

(11) Purity and Related Substances

Instrument: Agilent 1260 system with DAD detector or equivalent (Agilent Technologies, Santa Clara, CA, USA). Column: HALO 160 A C30 (4.6 × 150 mm, 2.7 µm; Advanced Materials Technology, Wilmington, DE, USA), P/N: 92114-730 or equivalent. Mobile phase A: 0.1% phosphoric acid in water. Mobile phase B: acetonitrile. Column temperature: 40 °C. Detection wavelength: 200 nm. Injection volume: 5 μL. Auto sampler temperature: 5 °C. Flow rate: 1 mL/min. Run time: 26 min. Gradient: 10–75% B (0–3 min), 75–95% B (3–20 min), 95–95% B (20–21 min), 95–10% B (21–21.1 min), 10–10% B (21.1–26 min). EK-16A was dissolved in acetonitrile to 1.0 mg/mL and diluted to a 0.05% sensitivity solution. Purity and related substances were calculated using the area normalization method.

(12) Chiral Purity

Instrument: Waters ACQUITY UPCC (Waters Corporation, Milford, MA, USA). Column: Regis (S,S) Whelk-O1 4.6 × 150 mm, 3.5 μm (Regis Technologies, Morton Grove, IL, USA). Detection wavelength: 215 nm. Column temperature: 35 °C. Injection volume: 7 μL. Sample tray temperature: 5 °C. Flow rate: 2.5 mL/min. Run time: 7 min. Mobile phase A: CO_2_. Mobile phase B: 0.1% isopropylamine in ethanol. Gradient: 10–10% B (0–0.5 min), 10–40% B (0.5–5.5 min), 40–40% B (5.5–6.5 min), 40–10% B (6.5–7.0 min). EK-16A was dissolved in ethanol to 2.0 mg/mL and diluted to a 0.05% sensitivity solution. Chiral purity was determined using the area normalization method.

(13) Residual Solvents

Instrument: An Agilent 7890A gas chromatograph (Agilent Technologies, Santa Clara, CA, USA) equipped with an Agilent 7697A headspace sampler (Agilent Technologies, Santa Clara, CA, USA) and a flame ionization detector (FID). Separation was performed on an Agilent DB-624 capillary column (Agilent Technologies, Santa Clara, CA, USA; 25 m × 0.2 mm I.D., 1.12 μm film thickness). Inlet temperature: 240 °C. Split ratio: 30:1. Carrier gas: nitrogen. Flow rate: 1.2 mL/min. Constant flow mode. Run time: 11.7 min. Detector temperature: 260 °C. Air flow: 400 mL/min. Hydrogen flow: 40 mL/min. Makeup N2 flow: 30 mL/min. Oven program: Hold at 45 °C for 0.2 min, ramp to 50 °C at 2 °C/min (hold 0.0 min), ramp to 250 °C at 35 °C/min (hold 3.3 min). Headspace parameters: Oven temperature 110 °C, loop temperature 150 °C, transfer line temperature 180 °C, GC cycle time 18 min, vial equilibration time 10 min, injection time 2.5 min, shake frequency 71 shakes/min, vial size 20 mL.

Reference standards were prepared in DMI at the following concentrations: methanol 3000 ppm, ethanol 5000 ppm, acetonitrile 410 ppm, n-hexane 290 ppm, ethyl acetate 5000 ppm (ICH guidelines). Sensitivity solution (LOQ): methanol 100 ppm, ethanol 99 ppm, acetonitrile 99 ppm, n-hexane 100 ppm, ethyl acetate 99 ppm. Sample preparation: 100 mg of EK-16A was weighed, transferred to a headspace vial, and 1 mL of DMI was added before capping.

Residual solvent results were calculated using the external standard method.

(14) Cellular Activity

The C11 cell line was constructed in-house by the laboratory of Professor Huanzhang Zhu, School of Life Sciences, Fudan University. It is an HIV-1 latently infected clonal Jurkat T-cell line with the virus integrated into the intron of RNPS1 and containing a green fluorescent protein (GFP) reporter gene under the control of the HIV-1 LTR.

Cryopreserved C11 cells were quickly thawed in a 37 °C water bath and transferred to a 15 mL centrifuge tube, to which ~5 mL of RPMI 1640 medium was added. After centrifugation at 800 rpm for 5 min, the medium was discarded. The cells were resuspended in RPMI 1640 medium containing 10% FBS and 1% penicillin/streptomycin, transferred to a 10 cm culture dish, and cultured at 37 °C with 5% CO_2_. After 24 h, the medium was replaced. Subsequently, cells were passaged and the medium was replaced every 48 h.

Expanded C11 cells were seeded into 96-well plates at a density of 4 × 10^5^ cells/mL (100 µL per well). EK-16A (dissolved in DMSO) was added to a final concentration of 10 nM, and the classic activator SAHA was used as a control at 1 µM. The untreated group was labeled Mock. After 48 h of culture (37 °C, 5% CO_2_), cells were collected into 1.5 mL EP tubes, centrifuged at 1000 rpm for 5 min, and the supernatant was discarded. Cells were washed with 300 µL PBS, centrifuged again, and resuspended in 300 µL PBS. The percentage of GFP-positive cells was determined using flow cytometry to evaluate the activation effect of each group.

#### 4.1.3. Stability Study Method for EK-16A

EK-16A compound was placed in glass vials, which were sealed and stored in a refrigerator (2~8 °C) and stability chambers (25 °C/60% RH, 40 °C/75% RH). Samples were taken at weeks 1 and 2 and months 1 and 3, and the content of EK-16A in them was analyzed using HPLC. Instrument: Agilent 1260 series HPLC system (Agilent Technologies, Santa Clara, CA, USA) with DAD detector. Column: Xbridge C18, 150 mm × 4.6 mm, 3.5 μm, (Waters Corporation, Milford, MA, USA). Mobile phase A: 0.1% TFA in water. Mobile phase B: 0.1% TFA in ACN. Gradient: 10–10% B (0–1.0 min), 10–95% B (1.0–10.0 min), 95–95% B (10.0–20.0 min), 95–10% B (20.0–20.1 min), 10–10% B (20.1–25 min). Flow rate: 1.2 mL/min. Injection volume: 10 μL. Sample concentration: 1 mg/mL. Diluent: MeOH. Column temperature: 40 °C. Wavelength: 210 nm. Autosampler temperature: 5 °C.

### 4.2. Development of EK-16A Self-Nanoemulsifying Drug Delivery System (SNEDDS)

#### 4.2.1. Permeability Test of EK-16A in Caco-2 Cells

Caco-2 cells (purchased from the American Type Culture Collection (ATCC), Manassas, VA, USA, Catalog No.: HTB-37) were cultured in MEM supplemented with 1% non-essential amino acids, 10% fetal bovine serum, 100 U/mL penicillin G, and 100 μg/mL streptomycin at 37.0 °C, 5.0% CO_2_, and saturated humidity. Cells were seeded onto Transwell 96-well plates (Corning Inc., Corning, NY, USA) at a density of 3.50 × 10^4^ cells/cm^2^ for transport experiments.

Receiver and transport buffer: Hank’s Balanced Salt Solution (HBSS from Gibco, Thermo Fisher Scientific, Waltham, MA, USA, pH 7.40 ± 0.05,) containing 10.0 mM HEPES (Sigma-Aldrich, Merck KGaA, Darmstadt, Germany).

Dosing solutions: EK-16A was prepared at 2.00 µM, digoxin at 10.0 µM, nadolol at 20.0 µM, and metoprolol at 20.0 µM, all diluted in transport buffer. Nadolol was used as a low-permeability control, metoprolol as a high-permeability control, and digoxin as an efflux transporter substrate.

Receiver and dosing solutions were added to appropriate wells of the Caco-2 cell plate, which was then incubated at 37.0 °C under 5.0% CO_2_ and saturated humidity for 120 min. Samples were collected from the donor and receiver compartments and analyzed by LC-MS/MS.

After the transport experiment, the integrity of the Caco-2 cell monolayer was assessed using the Lucifer Yellow Rejection Assay. Relative fluorescence unit (RFU) was measured using a microplate reader at excitation/emission wavelengths of 425/528 nm.

LC-MS/MS parameters: Instrument: Shimadzu LC-40D X3 (Shimadzu Corporation, Kyoto, Japan). Column: ACQUITY UPLC Protein BEH C4 300A 1.7 μm 2.1 × 50 mm (Waters Corporation, Milford, MA, USA). Internal standard: 250 nM labetalol. Mobile phase A: 0.1% formic acid in water. Mobile phase B: 0.1% formic acid in acetonitrile. Injection volume: 10 μL. Flow rate: 0.7 mL/min. Gradient: 5–95% B (0–0.40 min), 95–95% B (0.40–0.80 min), 95–5% B (0.80–0.81 min), 5–5% B (0.81–1.00 min). Mass spectrometer: SCIEX Triple Quad 6500 Plus (SCIEX, Framingham, MA, USA), ion source: ESI. EK-16A (positive mode): precursor ion *m/z* 645.4, product ion *m/z* 311.2. Labetalol (positive mode): precursor ion *m/z* 329.2, product ion *m/z* 162.1.

Chromatograms for analytes and internal standard were acquired using Analyst software (version 1.6.3, SCIEX, Framingham, MA, USA), and data were processed using Multi Quant software (version 3.0, SCIEX, Framingham, MA, USA).

#### 4.2.2. Solubility of EK-16A

Approximately 2 mg of EK-16A was added to 0.1 mL of various vehicles: Labrasol ALF (Gattefossé, Saint-Priest, France), Labrafac MC60 (Gattefossé, Saint-Priest, France), Capryol 90 (Gattefossé, Saint-Priest, France), Maisine CC (Gattefossé, Saint-Priest, France), corn oil (Sigma-Aldrich, St. Louis, MO, USA), soybean oil (Sigma-Aldrich, St. Louis, MO, USA), 30% HP-β-CD aqueous solution (Sigma-Aldrich, St. Louis, MO, USA), and 30% SBE-β-CD aqueous solution (CyDex Pharmaceuticals, Lenexa, KS, USA). The mixtures were shaken (25 °C, 700 rpm). After 24 h, the appearance was recorded, and the samples were centrifuged. The supernatant was collected, and solubility was determined by HPLC.

#### 4.2.3. Formulation Screening Procedure 

Fifteen blank formulations (without EK-16A) were designed by mixing the excipients in the specified proportions using a magnetic stirrer, and the appearance of each mixture was recorded. Each formulation was then diluted 100-fold with purified water and stirred at 37 °C and 100 rpm to allow complete emulsification; the performance of which was evaluated.

Based on the initial screening, three formulations with unsatisfactory appearance and emulsification were discarded. The remaining 12 formulations were re-prepared as drug-loaded formulations by adding EK-16A preferentially to the excipient that exhibited the highest solubility for the compound. The appearance of each drug-loaded formulation was again observed.

The preparation of formulation #A-1 is described as a representative example. EK-16A (20 mg) and Maisine CC (Gattefossé, Saint-Priest, France) (3.0 g) were weighed into a glass bottle and stirred at 700 rpm for 30 min until complete dissolution. Cremophor ELP (BASF, Ludwigshafen, Germany) (3.2 g), preheated to a liquid state, was added and the mixture was stirred at 700 rpm for 15 min to obtain a homogeneous system. Soybean oil (Hunan Erkang Pharmaceutical Co., Ltd., Changsha, Hunan, China) (3.0 g) and ethanol (Merck KGaA, Darmstadt, Germany) (0.8 g) were then added, and stirring was continued at 700 rpm for another 15 min until a uniform mixture was obtained. The total weight of the final SNEDDS-EK-16A formulation was 10.02 g.

The emulsification behavior of the formulations under simulated gastrointestinal conditions was evaluated as follows: An aliquot (100 μL) of each formulation was slowly added to 900 μL of preheated FaSSGF (fasted state simulated gastric fluid, from Biorelevant.com Ltd., London, UK) and shaken at 37 °C and 300 rpm. After 30 min, the emulsification effect was recorded, and the particle size (D90) and polydispersity index (PDI) were measured. Subsequently, 200 μL of the resulting FaSSGF mixture was transferred into 600 μL of preheated FaSSIF (fasted state simulated intestinal fluid, from Biorelevant.com Ltd., London, UK) and shaken at 37 °C and 300 rpm for 120 min. The emulsification effect, D90, and PDI were again determined.

Particle size (D90) and polydispersity index (PDI) of the SNEDDS formulations were measured using a Malvern Zeta sizer Nano ZEN3600 (Malvern Panalytical, Malvern, UK) based on dynamic light scattering (DLS). The measurements were performed at a fixed scattering angle of 173° with a helium–neon laser (wavelength 633 nm) at 25 °C. The formulation was appropriately diluted with purified water before measurement. After an equilibration time of 60 s, each sample was measured three times, with each measurement consisting of 10 runs of 10 s duration.

The PDI was directly obtained from the cumulant analysis of the intensity autocorrelation function. To obtain the D90 value, the intensity-weighted size distribution was converted into a volume-weighted size distribution using Mie theory, with the refractive indices of the sample and dispersant properly set. The General Purpose algorithm (non-negative least squares, NNLS) embedded in the Zeta sizer software (version 8.02) was used for the inversion of the correlation function. The D90 (Dv (90)) was then read from the cumulative volume distribution as the size below which 90% of the total droplet volume lies.

#### 4.2.4. Stability Study of EK-16A SNEDDS Formulations

EK-16A raw material formulations A#1, A#8, A#11, and A#12 were weighed, sealed, and stored in a refrigerator (2~8 °C) and stability chambers (25 °C/60% RH, 40 °C/75% RH). Samples were taken at weeks 1 and 2 and months 1 and 3, and the appearance was observed. After dilution (tetrahydrofuran: methanol = 1:1, *v*/*v*), the EK-16A content in each formulation was determined by HPLC. Instrument: Agilent 1260 series HPLC system (Agilent Technologies, Santa Clara, CA, USA) with DAD detector. Column: Xbridge C18, 150 mm × 4.6 mm, 3.5 μm, (Waters Corporation, Milford, MA, USA). Mobile phase A: 0.1% TFA in water. Mobile phase B: 0.1% TFA in ACN. Gradient: 10–10% B (0–1.0 min), 10–95% B (1.0–20.0 min), 95–10% B (20.0–20.1 min), 10–10% B (20.1–25.0 min). Flow rate: 1.2 mL/min. Injection volume: 10 μL. Standard concentration: 1 mg/mL. Column temperature: 40 °C. Wavelength: 210 nm. Autosampler temperature: 5 °C.

### 4.3. In Vivo Pharmacokinetics and Metabolism of EK-16A SNEDDS

#### 4.3.1. In Vivo Bioavailability of EK-16A SNEDDS

Preparation of dosing formulations. For the intravenous (IV) injection group, EK-16A was dissolved in a 1:1 (*v*/*v*) mixture of 50% ethanol and 50% Cremophor EL to obtain a 0.1 mg/mL stock solution, which was further diluted with saline to 0.005 mg/mL. The IV dose was 35 μg/kg. For the oral gavage groups, EK-16A compound was first dissolved in DMSO to prepare a 10 mg/mL solution and then diluted with saline to 0.5 mg/mL (oral dose 10 mg/kg). The SNEDDS formulation (#A-1) was diluted with purified water and vortexed for 3 min to yield a 0.5 mg/mL solution, also administered at 10 mg/kg.

Animal experiments and sample collection. All procedures involving animals were approved by the Institutional Animal Care and Use Committee (IACUC) of WuXi AppTec (Nanjing) Co., Ltd. (approval No. NJ-20240522-Rats). Male SD rats weighing 242.36–298.28 g were used. For the EK-16A raw material and IV injection groups (n = 3 per group), blood samples were collected pre-dose (0 h) and at 0.0042 h (15 s), 0.083, 0.25, 0.5, 1, 2, 8, and 24 h post-dose. For the SNEDDS #A-1 oral gavage group (n = 4), blood sampling was performed pre-dose (0 h) and at 0.5, 1, 2, 4, 8, 12, 24, 32, 48, 56, and 72 h post-dose.

Whole blood (approximately 0.2 mL) was collected via jugular vein puncture into commercial tubes containing K_2_-EDTA and immediately placed on wet ice. Within 60 min of collection, samples were centrifuged at 3200× *g* for 10 min at 4 °C. The resulting plasma supernatant was divided into two aliquots, quickly frozen on dry ice, and stored at –60 °C: one aliquot was used for LC-MS/MS determination of EK-16A concentration, and the other for metabolite analysis. Excreta (feces and urine) were collected in 50 mL centrifuge tubes and stored at −60 °C for subsequent metabolite identification.

Calibration curve and quality control. A calibration curve for EK-16A was constructed using eight non-zero standards in the concentration range 0.500–500 ng/mL (0.5, 1, 5, 25, 50, 100, 250, and 500 ng/mL). Linear regression was performed, and the correlation coefficient was calculated. Precision and accuracy were evaluated at the lower limit of quantitation (LLOQ, 1.50 ng/mL), and at low (20.0 ng/mL), medium (200 ng/mL), and high (400 ng/mL) concentrations, each injected six times. During the LC-MS/MS analysis of plasma samples, quality control (QC) samples at these four concentrations were included in each sequence to ensure instrument performance. All QC samples met the acceptance criteria, with accuracy (Bias%) within ±20% of the nominal value.

Biological sample processing. An aliquot (20 µL) of each sample was mixed with 60 µL of internal standard solution (containing 100 ng/mL each of labetalol, tolbutamide, verapamil, dexamethasone, glyburide, and celecoxib in 0.1% formic acid in acetonitrile) for protein precipitation. The mixture was vortexed at 800 rpm for 10 min and then centrifuged at 3220× *g* for 15 min at 4 °C. The supernatant (55 µL) was transferred to a clean 96-well plate and centrifuged again at 3220× *g* for 5 min at 4 °C before LC-MS/MS analysis.

LC-MS/MS conditions. Chromatographic separation was performed on an ACQUITY UPLC System (Waters Corporation, Milford, MA, USA) equipped with an ACQUITY UPLC Protein BEH C4 column (300 Å, 1.7 µm, 2.1 × 50 mm; Waters Corporation, Milford, MA, USA). The injection volume was 5.0 μL. Mobile phase A consisted of water/acetonitrile (95:5, *v*/*v*) containing 0.1% ammonia, and mobile phase B consisted of water/acetonitrile (5:95, *v*/*v*) containing 0.1% ammonia. The gradient program was as follows: 10% B for 0–0.3 min, 10→100% B from 0.3 to 0.9 min, 100% B from 0.9 to 1.5 min, 100→10% B from 1.5 to 1.51 min, and 10% B from 1.51 to 1.6 min. The flow rate was 0.65 mL/min. Mass spectrometry was performed on an API5000 instrument in negative electrospray ionization mode with multiple reaction monitoring (MRM). The ion pair for EK-16A was *m/z* 643.5→114.9 (retention time 1.13 min), and the internal standard (celecoxib) was monitored at *m/z* 380.0→316.0 (retention time 0.90 min).

Data processing. Chromatogram acquisition and integration were performed using Analyst 1.6.3 software. Pharmacokinetic parameters were calculated from plasma concentration–time data using a non-compartmental model in WinNonlin version 8.3.5 (Certara, Princeton, NJ, USA). The linear-log trapezoidal rule was applied to calculate the area under the curve (AUC). Theoretical sampling times and doses were used for all calculations.

#### 4.3.2. Study of In Vivo Metabolites and Metabolic Pathways of EK-16A

Plasma and excreta (feces and urine) samples from rats in the IV and A#1 formulation oral gavage groups described in Section 4.3.1 were used for metabolite and metabolic pathway analysis. Samples were retrieved from −60 °C storage and allowed to warm to room temperature. For plasma, two volumes of acetonitrile were added, the mixture was centrifuged at 14,000 rpm for 10 min, and the supernatant was analyzed by LC-TOFMS. For excreta, an equal volume of purified water (5 mL) was added and vortexed for 2 min; then, 7 mL of dichloromethane was added, vortexed for 2 min, and centrifuged. The lower (dichloromethane) layer was collected and dried under nitrogen. The residue was dissolved in 100 μL of acetonitrile and centrifuged at 14,000 rpm for 10 min, and the supernatant was analyzed by LC-TOFMS.

Instrument: Bruker tims TOF Pro2 (Bruker Corporation, Billerica, MA, USA). Column: Bruker Intensity solo 2, 100 × 20 mm. Mobile phases: purified water and acetonitrile. Flow rate: 0.3 mL/min. Gradient: 20% acetonitrile held for 0.5 min, increased to 100% acetonitrile by 10 min, held for 2 min, then returned to 20% acetonitrile at 12.1 min and held until 15 min. Injection volume: 10 μL. Ion source: VIP-HESI, negative ion mode. Data acquisition mode: PASEF. Data processing software: Bruker MetaboScape 2024b.

### 4.4. Study of PI3K-AKT Signaling Pathway of EK-16A in a SHIV Latently Infected Rhesus Macaque Model

Plasma samples were collected from control, model, and EK-16A treatment groups of rhesus macaques. In the treatment group, macaques received an oral EK-16A formulation (A#1) every 2 days at a dose of 4 mg/kg, for a total of 5 doses, administered after meals. Blood samples were collected from six SHIV latently infected rhesus macaques via EDTA venous puncture before treatment (0 d) and on day 8 after the treatment commencement. Three healthy rhesus macaques also had 1 mL of blood collected via EDTA venous puncture. Plasma was obtained by centrifugation, heat-inactivated at 56 °C for 30 min, and stored at −80 °C.

A total of 15 plasma samples were analyzed: Healthy control group (Control): JI, J2, J3. SHIV-infected latent model group (Model): A3-0d, A5-0d, B5-0d, C2-0d, WB6-0d, WD3-0d. EK-16A-treated group (EK-16A): A3-8d, A5-8d, B5-8d, C2-8d, WB6-8d, WD3-8d.

The animals were housed in a biosafety level 3 (ABSL-3) facility. The animal study protocol for rhesus macaques (SHIV-infected model) was approved by the Institutional Animal Care and Use Committee (IACUC) of the Chinese Academy of Medical Sciences (CAMS) (protocol code XJ23001, date of approval: 14 March 2023).

#### 4.4.1. Metabolomics Study of EK-16A in SHIV Latently Infected Rhesus Macaques

For metabolomics analysis, 200 µL of each heat-inactivated plasma sample was vortexed for 10 s. Proteins were precipitated by adding 800 µL of methanol:acetonitrile (2:1, *v*/*v*) and vortexing for 1 min. The samples were then sonicated in an ice-water bath for 10 min and allowed to stand at –20 °C for 4–6 h. After centrifugation at 12,000 rpm for 10 min at 4 °C, the entire supernatant was transferred to a new 10 mL EP tube and dried under a stream of nitrogen. The residue was reconstituted in 300 µL of methanol:water (1:1, *v*/*v*), vortexed for 1 min, and sonicated again in an ice-water bath for 10 min. The reconstituted solution was transferred to a 1.5 mL centrifuge tube and centrifuged at 12,000 rpm for 10 min at 4 °C. A volume of 150 µL of the final supernatant was transferred to an LC-MS vial with an insert, and 5 µL of mixed internal standard (0.1 mg/mL) was added prior to analysis. Quality control (QC) samples were prepared by mixing equal volumes of extracts from all samples.

Chromatographic separation was performed on a Waters ACQUITY UPLC I-Class Plus system coupled to a Thermo QE HF high-resolution mass spectrometer. The column was an ACQUITY UPLC HSS T3 (100 mm × 2.1 mm, 1.8 μm) maintained at 45 °C. Mobile phase A was water containing 0.1% formic acid, and mobile phase B was acetonitrile. The flow rate was 0.35 mL/min, and the injection volume was 5 μL. The gradient program was as follows: 5% B for 0.0–2.0 min, 5→30% B from 2.0 to 4.0 min, 30→50% B from 4.0 to 8.0 min, 50→80% B from 8.0 to 10.0 min, 80→100% B from 10.0 to 14.0 min, 100% B from 14.0 to 15.0 min, 100→5% B from 15.0 to 15.1 min, and 5% B from 15.1 to 16.0 min. The mass range was *m/z* 70–1050.

Metabolomics data were acquired using the UPLC-HRMS system. Metabolites in rhesus macaque plasma were identified and quantified by searching multiple databases (HMDB, Lipidmaps v2.3, METLIN, and LuMet-Animal 3.0) based on accurate mass, MS/MS fragments, retention time, and isotope distribution.

#### 4.4.2. Proteomics Study of EK-16A in SHIV Latently Infected Rhesus Macaques

Fifteen plasma samples were processed using a low-abundance protein enrichment kit (Oebiotech) for magnetic nanobead-based protein enrichment, digestion, and desalting.

Protein enrichment. Magnetic nanomaterial suspension (5 µL) was placed in a 1.5 mL centrifuge tube, mixed with 75 µL of dilution buffer, vortexed for 10–30 s, and subjected to magnetic separation; the supernatant was discarded. The beads were resuspended in 50 µL of dilution buffer, and 25 µL of plasma was added. The mixture was incubated in a thermomixer at 37 °C and 1000 rpm for 30 min. After magnetic separation, the beads were washed three times with 75 µL of dilution buffer at 26 °C and 1000 rpm for 5 min each wash. The captured proteins on the beads were used for the subsequent digestion.

Protein digestion. The washed beads were resuspended in 25 µL of digestion buffer, and 0.5 µL of reconstituted trypsin (0.5 µg/µL in ultrapure water) was added. The mixture was incubated in a thermomixer at 37 °C and 1000 rpm for 4 h (minimum 3 h). After brief centrifugation (2 s), the supernatant was collected by magnetic separation into a new 1.5 mL tube. Loading buffer (25 µL) was added to each sample and mixed thoroughly at 26 °C for 5 min. Then, 12.5 µL of stop buffer was added, which produced a small amount of white precipitate; the mixture was again mixed thoroughly at 26 °C for 5 min until the precipitate disappeared.

Peptide desalting. All liquid samples were loaded onto a desalting column and centrifuged at 1000× *g* for 3–5 min. Subsequently, 25 µL of wash buffer 1 was loaded, and the column was centrifuged under the same conditions. The same step was repeated with 25 µL of wash buffer 2. A new collection tube was then placed, and 25 µL of elution buffer was loaded; the column was centrifuged at 1000× *g* for 3–5 min at room temperature. The eluted peptides were dried down and reconstituted in 8 µL of LC-MS-grade mobile phase A, followed by vortexing for 15–20 min before analysis.

Internal standard and mass spectrometry conditions. The iRT standard (Biognosys), a kit containing 11 synthetic peptides not found in nature, was used as an internal standard due to its stability, sensitivity, and optimized retention times. iRT was mixed with the samples at a 1:20 (*v*/*v*) ratio.

Mass spectrometry data were acquired using a Bruker tims TOF HT mass spectrometer in data-independent acquisition (DIA) mode. The column was a 15 cm × 100 µm, 1.9 µm C18 column. Mobile phase A was 0.1% formic acid in water, and mobile phase B was 0.1% formic acid in acetonitrile. The gradient was as follows: 6→28% B from 0.0 to 22.5 min, 28→80% B from 22.5 to 24.0 min, and 80% B from 24.0 to 25.0 min. The flow rate was not specified in the original (placeholder “μL/min”), so it has been omitted. The mass range was *m/z* 300–1500.

Data processing. DIA raw data were processed using Spectronaut Pulsar 18.7 software for protein identification and quantification. The search database was the UniProt Macaca mulatta reference proteome (taxon ID 9544, version 2024.4.10).

#### 4.4.3. Molecular Docking Network Experiment of EK-16A with Potential In Vivo Targets

(1) Three-Dimensional Structures of EK-16A and Targets

Based on the structure of EK-16A, the SMILES string (CCCCCCCCCCCC(O[C@@]12CC@@H[C@]3(C=C(C)[C@@H]4OC(C(C)C(C)C)=O)[C@]4(O)C@HC(CO)=CC@@([H])C1C2(C)C)=O) was generated using Chem Draw. The ligand.pdbqt file (3D structure of the ligand) was generated using obabel. Based on the Uni Prot ID of potential targets, the protein.pdb file was downloaded from Alpha Fold. The protein.pdbqt file (3D structure of the target) was generated using Auto Dock Tools (version 4.2).

(2) Molecular Docking Parameters

Before docking, the receptor.gpf file under the config directory was generated using Auto Dock Tools. This file contains parameters for molecular docking, including npts, spacing, receptor_types, ligand_types, grid center, smooth, map protein, and dielectric.

(3) Molecular Docking Analysis

Auto Dock Vina software (version 1.2.7)—a widely used molecular docking software for studying the binding between proteins and small molecules—was used to perform the docking calculation between the ligand.pdbqt and protein.pdbqt files, generating the ligand_out.pdbqt file. The output includes the coordinates and interaction energies of the best binding modes.

(4) Interaction Analysis

Protein–Ligand Interaction Profiler (PLIP, version 2.4.0) is a software tool for predicting and analyzing interactions between proteins and small molecule ligands. It predicts interactions based on factors such as hydrogen bonds, hydrophobic interactions, and ionic pairs. PLIP was used with the ligand_out.pdbqt and protein.pdb files to generate a report.txt file in the plip directory, containing the results of protein–small molecule ligand interactions.

(5) Visualization of Molecular Docking

PyMOL software (version 3.1) was used to visualize the 3D structure of the EK-16A-protein complex from four different angles. The interactions between EK-16A and the protein were also visualized.

## 5. Conclusions

Supercritical fluid technology was applied to extract the EK-16A compound from *Euphorbia kansui*, resulting in an extraction process with high yield and high purity that holds promise for scaled-up production.

EK-16A is a BCS Class IV compound with low solubility and permeability and poor stability at room temperature. Using SNEDDS technology significantly enhances its stability and enables its successful oral delivery into the systemic circulation.

Through proteomics experiments, we found that EK-16A significantly downregulates the PI3K/Akt signaling pathway. However, further experiments are needed to confirm this conclusion.

## Figures and Tables

**Table 1:** Physicochemical properties and quality standard results for EK-16A compound.

No.	Analysis Item	Result
1	Appearance	White solid
2	Solubility	Insoluble in water
3	NMR (^1^H, ^13^C, DEPT, COSY, NOESY, HSQC-DEPT, HMBC, TOCSY)	Consistent with theoretical structure
4	High-Resolution Mass Spectrometry (HRMS)	Exact Mass: 644.4288. Acquired Mass: 645.4365 [M + H]^+^
5	Infrared Spectroscopy (IR)	O-H: 3447.9 cm^−1^; C=O: 1725.7 cm^−1^; C-O-C: 1175.2 cm^−1^ and 1023.0 cm^−1^
6	Ultraviolet Spectroscopy (UV)	λ = 194.0 nm; λ = 289.5 nm
7	Elemental Analysis (EA)	Consistent with theoretical structure
8	Purity and Related Substances (HPLC)	Purity: 98.0%. Single impurity: RRT 0.981 (0.64%), RRT 1.019 (1.2%), RRT 1.083 (0.15%). Total impurities: 2.0%
9	Chiral Purity and Enantiomers (SFC)	Chiral purity: 100.00%
10	Water Content (Karl Fischer)	0.20%
11	Residual Solvents (GC)	Acetonitrile: <LOQ (LOQ 99 ppm). Methanol: <LOQ (LOQ 100 ppm). Ethanol: <LOQ (LOQ 99 ppm). Ethyl acetate: <LOQ (LOQ 99 ppm). n-Hexane: <LOQ (LOQ 100 ppm)
12	Optical Rotation (OR)	[α]D^20^ = −6.39
13	Crystal Form (XRPD)	Amorphous
14	Differential Scanning Calorimetry (DSC)	Two melting ranges: 122.42~194.24 °C; 223.57~271.03 °C
15	Thermogravimetric Analysis (TGA)	Weight loss of 0.04% from 40 °C to 150 °C; weight loss of 86% from 150 °C to 800 °C
16	Cellular Activity (Flow Cytometry)	Activation of GFP reporter gene in C11 cells by 10 nM EK-16A: 86.6%
17	Content of Assay (QNMR)	96.88%

**Table 2:** Stability results for EK-16A compound.

Storage Condition	Initial	1 Week	2 Week	1 Month	3 Months
2~8 °C	100%	102.0%	98.8%	101.3%	99.4%
25 °C/60% RH	100%	101.0%	99.9%	100.1%	49.8%
40 °C/75% RH	100%	86.8%	64.7%	36.4%	2.4%

**Table 3:** Solubility results for EK-16A.

Excipient	Temperature (°C)	Equilibrium Solubility (mg/mL)
Labrasol ALF	37 °C	10.10
Labrafac MC60		17.49
Capryol 90		17.41
Maisine CC		20.14
Corn oil		17.76
Soybean oil		18.35
30% HP-β-CD aqueous		0.39
30% SBE-CD aqueous		0.08

**Table 4:** Formulation design of EK-16A-SNEDDS.

Formulation (*w*/*w* %)	#A-1	#A-2	#A-3	#A-4	#A-5	#A-6	#A-7	#A-8	#A-9	#A-10	#A-11	#A-12	#A-13	#A-14	#A-15
EK-16A	0.2	0.2	0.2	0.2	0.2	0.2	0.2	0.2	0.2	0.2	0.2	0.2	0.2	0.2	0.2
Soybean oil	30														
Maisine CC	30			25	30		40	30					30	25	20
Cremophor ELP	32												32	42	52
Plurol CC 497		13													
Labrafac Lipophile WL 1349		10													
Capryol 90			30		20	20					50				
Corn oil						30									
Capmul MCM									12	30		20	30	25	20
Tween 80			70	55											
PEG 400				20						20		8			
Labrasol ALF		77			30	30	60	70							
VE-TPGS									28	50					
Kolliphor RH40											50	72			
Ethanol	8				20	20							8	8	8
Water									60						

**Table 5:** Formulation evaluation of SNEDDS (blank formulation).

Item	#A-1	#A-2	#A-3	#A-4	#A-5	#A-6	#A-7	#A-8	#A-9	#A-10	#A-11	#A-12	#A-13	#A-14	#A-15
Initial Appearance	Clear	Clear	Clear	Clear	Clear	Clear	Clear	Clear	Semi-solid	Paste	Clear	Clear	Clear	Clear	Clear
Emulsification (Water)	A	C	B	B	D	D	E	D	/	/	A	A	A	A	A

**Table 6:** Formulation evaluation of EK-16A-SNEDDS (drug-loaded).

Item	#A-1	#A-2	#A-3	#A-4	#A-5	#A-6	#A-8	#A-11	#A-12	#A-13	#A-14	#A-15
Appearance	Clear	Clear	Clear	Clear	Clear	Clear	Clear	Clear	Clear	Clear	Clear	Clear
Emulsification (gastric fluid)	A	E	E	C	E	E	C	C	A	E	A	A
Particle Size D90 nm (gastric fluid)	71.3	/	/	1280	/	/	813	288	741	/	648	711
PDI (gastric fluid)	0.090	/	/	0.484	/	/	0.657	0.295	0.284	/	/	/
Emulsification (intestinal fluid)	A	/	/	C	/	/	C	C	A	/	A	A
Particle Size D90 nm (intestinal fluid)	68.6	/	/	881	/	/	347	396	340	/	281	285
PDI (intestinal fluid)	0.082	/	/	0.484	/		0.232	0.439	0.166	/	/	/

**Table 7:** Summary of physical appearance and assay% stability results for EK-16A compound and oral formulations under 2~8 °C, 25 °C/60% RH, and 40 °C/75% RH conditions.

Condition	Time Point	EK-16A	#A-1	#A-8	#A-11	#A-12
	Initial	White Powder, 100.0	Clear, 101.5	Clear, 102.0	Clear, 94.9	Clear, 93.4
2~8 °C	1 Week	Caked, 102.0	Clear, 108.3	Clear, 103.3	Solidified, 96.4	Solidified, 94.6
	2 Weeks	Caked, 98.8	Clear, 105.0	Clear, 101.7	Solidified, 92.4	Solidified, 91.8
	1 Month	Caked, 101.3	Clear, 104.9	Clear, 102.6	Solidified, 89.2	Solidified, 89.8
	3 Months	Caked, 99.4	Clear, 106.6	Clear, 101.8	Solidified, 79.3	Solidified, 80.7
25 °C/60% RH	1 Week	Melted, 101.0	Clear, 107.5	Clear, 104.0	Clear, 85.5	Solidified, 86.5
	2 Weeks	Melted, 99.9	Clear, 105.7	Clear, 102.1	Clear, 79.7	Solidified, 78.6
	1 Month	Melted, 100.1	Clear, 104.8	Clear, 101.5	Clear, 70.4	Solidified, 68.4
	3 Months	Melted, 49.8	Clear, 105.2	Clear, 99.8	Clear, 41.2	Solidified, 38.0
40 °C/75% RH	1 Week	Melted, 86.8	Clear, 108.4	Clear, 104.3	Clear, 69.6	Clear, 65.5
	2 Weeks	Melted, 64.7	Clear, 107.8	Clear, 107.7	Clear, 53.3	Clear, 47.1
	1 Month	Melted, 36.4	Clear, 105.2	Clear, 100.1	Clear, 24.7	Clear, 21.7
	3 Months	Melted, 2.4	Clear, 106.7	Clear, 97.0	Clear, 6.4	Clear, 7.9

**Table 8:** Pharmacokinetic parameters of EK-16A.

Parameter	EK-16A Injection	EK-16A Oral Formulation (#A-1)
Administration Route	IV Bolus	Oral Gavage
Dose (mg/kg)	0.035	10
Cmax (ng/mL)	500	9.58
Tmax (h)	--	24.0
AUC0-last (ng·h/mL)	163	193
Bioavailability (%)	--	0.445
Dose (mg/kg)	0.035	10

Note: -- indicates not applicable.

**Table 9:** Summary of metabolites (1–6) and metabolic pathway information.

No.	Source	Metabolic Pathway	Metabolite Name	Metabolite Structure
1	Rat plasma (injection) Rat plasma (oral #A-1) Rat excreta (oral #A-1)	Carboxylesterase EC 3.1.1.1 hydrolysis	13-O-dodecanoylingenol	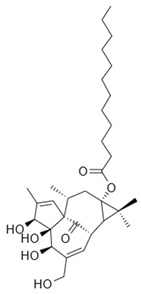
2	Rat excreta (oral #A-1)	Carboxylesterase EC 3.1.1.1 hydrolysis	3-O-(2,3-dimethylbutanoyl)ingenol	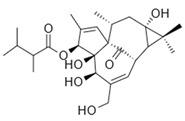
3	Rat plasma (oral #A-1) Rat excreta (oral #A-1)	Carboxylesterase EC 3.1.1.1 hydrolysis	Ingenol	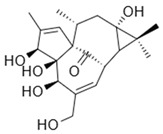
4	Rat excreta (oral #A-1)	Sulfotransferase EC 2.8.2.2, sulfonation	3-O-(2,3-Dimethylbutanoyl)-13-O-dodecanoyl-20-O-sulfoingenol	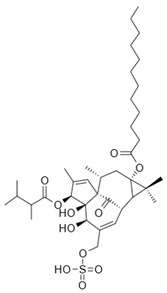
5	Rat excreta (oral #A-1)	Carboxylesterase EC 3.1.1.1 hydrolysis; Sulfotransferase EC 2.8.2.2 sulfonation	3-O-(2,3-Dimethylbutanoyl)-20-O-sulfoingenol	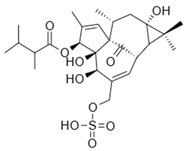
6	Rat excreta (oral #A-1)	Carboxylesterase EC 3.1.1.1 hydrolysis; Sulfotransferase EC 2.8.2.2 sulfonation	13-O-Dodecanoyl-20-O-sulfoingenol	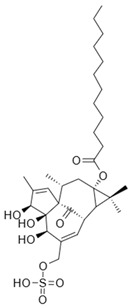

**Table 10:** Statistical table of differentially expressed proteins.

Group Comparison	Total DEPs	Upregulated	Downregulated
EK-16A vs. Model	233	123	110
Model vs. Control	418	332	86

**Table 11:** Names and amino acid residue interaction sites for molecules showing affinity for EK-16A.

No.	Name	Interaction
1	Tyrosine-protein kinase (LOC114669853)	Hydrophobic interactions: PRO297, VAL300, THR322, THR324, ASP325, PRO496, TYR582, TYR634, TYR638, ASP641, LYS642, GLU959. Hydrogen bonds: GLU298, ARG323, THR324, ASP325, LYS886, LYS965. Salt bridge: LYS642, HIS963.
2	Laminin subunit alpha-1 Fragment (EGK_09538)	Hydrophobic interactions: ARG283, HIS289, PRO292, TRP293, TYR318. Hydrogen bonds: TRP293, TYR318. Salt bridge: HIS289.
3	Insulin-like growth factor I (IGF1)	Hydrophobic interactions: ALA61, PHE64, VAL65, LEU102, LEU105, GLU106. Hydrogen bonds: ARG69, GLU106.

## Data Availability

The data reported in this paper have been deposited in OMIX, China National Center for Bioinformation/Beijing Institute of Genomics, Chinese Academy of Sciences (https://ngdc.cncb.ac.cn/omix) accession no. OMIX016029 (accessed on 5 April 2026).

## Abbreviations

The following abbreviations are used in this manuscript:

AIDS	Acquired Immunodeficiency Syndrome
ART	Antiretroviral Therapy
AUC	Area Under the Curve
BCS	Biopharmaceutics Classification System
DEP	Differentially Expressed Protein
DIA	Data-Independent Acquisition
DMSO	Dimethyl Sulfoxide
EC	Enzyme Commission
ER	Efflux Ratio
ESI	Electrospray Ionization
FaSSGF	Fasted State Simulated Gastric Fluid
FaSSIF	Fasted State Simulated Intestinal Fluid
FBS	Fetal Bovine Serum
FC	Fold Change
GC	Gas Chromatography
GFP	Green Fluorescent Protein
HIV	Human Immunodeficiency Virus
HPLC	High-Performance Liquid Chromatography
HRMS	High-Resolution Mass Spectrometry
IACUC	Institutional Animal Care and Use Committee
IR	Infrared Spectroscopy
IS	Internal Standard
IV	Intravenous
KEGG	Kyoto Encyclopedia of Genes and Genomes
LC-MS/MS	Liquid Chromatography–Tandem Mass Spectrometry
LC-TOFMS	Liquid Chromatography–Time of Flight Mass Spectrometry
LLOQ	Lower Limit of Quantitation
LRA	Latency-Reversing Agent
MEM	Minimum Essential Medium
MRM	Multiple Reaction Monitoring
NMR	Nuclear Magnetic Resonance
OR	Optical Rotation
Papp	Apparent Permeability Coefficient
PBS	Phosphate-Buffered Saline
PDI	Polydispersity Index
PKC	Protein Kinase C
QC	Quality Control
QNMR	Quantitative Nuclear Magnetic Resonance
RH	Relative Humidity
RT	Retention Time
SFC	Supercritical Fluid Chromatography
SHIV	Simian-Human Immunodeficiency Virus
SNEDDS	Self-Nanoemulsifying Drug Delivery System
TGA	Thermogravimetric Analysis
UPLC	Ultra-Performance Liquid Chromatography
